# Mental Well‐Being Is Associated With Temporomandibular Disorder Pain Onset and Remission

**DOI:** 10.1111/joor.70236

**Published:** 2026-06-18

**Authors:** S. Vallin, P. Liv, B. Häggman‐Henrikson, C. M. Visscher, F. Lobbezoo, A. Lövgren

**Affiliations:** ^1^ Department of Odontology, Orofacial Pain and Jaw Function, Faculty of Medicine Umeå University Umeå Sweden; ^2^ Department of Public Health and Clinical Medicine, Section of Sustainable Health Umeå University Umeå Sweden; ^3^ Department of Orofacial Pain and Jaw Function, Faculty of Odontology Malmö University Malmö Sweden; ^4^ Department of Orofacial Pain and Dysfunction, Academic Centre for Dentistry Amsterdam (ACTA) University of Amsterdam and Vrije Universiteit Amsterdam Amsterdam the Netherlands

**Keywords:** cohort studies, orofacial pain, quality‐of‐life, social determinants

## Abstract

**Background:**

Temporomandibular disorders (TMD) impact the everyday lives of those affected. However, the longitudinal role of sick leave and health‐related quality of life (HRQoL) in TMD‐pain onset and remission is largely unknown.

**Objectives:**

To explore whether TMD‐pain onset and remission are associated with HRQoL and sick‐leave history.

**Methods:**

The cohort comprised 33 571 individuals, aged 23–74 years, who participated in a community intervention programme and had routine dental check‐ups in Västerbotten County, Sweden. HRQoL was assessed using two domains, i.e., Mental Component Summary (mental well‐being) and Physical Component Summary (physical well‐being), of the Short Form 36 Health Survey Questionnaire. Sick‐leave history was defined as self‐reported sick leave for at least 6 months. Associations with TMD‐pain onset and remission were analysed using Markov multi‐state models.

**Results:**

Better mental and physical well‐being were associated with lower rates of TMD‐pain onset (hazard ratio [HR] per 10‐unit increase: 0.84, CI: 0.79–0.89, and 0.90, CI: 0.84–0.96, respectively), whereas sick leave was associated with higher rates (HR: 1.18, CI: 1.03–1.36). Better mental and physical well‐being were associated with higher rates of TMD‐pain remission (HR per 10‐unit increase: 1.33, CI: 1.28–1.37, and 1.68, CI: 1.62–1.75, respectively), whereas sick leave was associated with lower rates (HR: 0.49, CI: 0.46–0.53).

**Conclusions:**

The associations of sick leave, mental well‐being, and physical well‐being with TMD‐pain remission emphasise the importance of applying a holistic approach to pain assessment and management. In addition, mental well‐being is associated with TMD‐pain onset, indicating that this could be a factor to consider when designing preventative strategies.

## Graphical abstract

In this cohort study of 33,571 adults, individuals with better physical and mental well‐being were less likely to report temporomandibular disorder pain onset and more likely to report remission. In contrast, individuals with a history of sick leave were more likely to report onset and less likely to report remission.

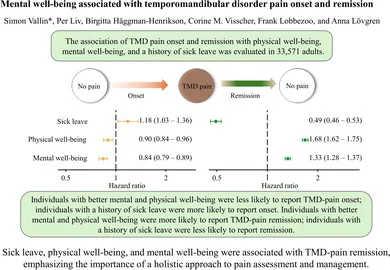

## 1 Introduction

Of the pain conditions managed in dentistry, temporomandibular disorders (TMD) are the most common reason for chronic orofacial pain, affecting approximately 10% of the general adult population [1, 2]. TMD symptoms can include both pain symptoms related to the temporomandibular joint, masticatory muscles, and surrounding structures as well as restricted jaw function [3, 4]. These symptoms commonly interfere with daily jaw functions such as talking and eating, and negatively affect the quality of life [5]. In common with many other chronic pain conditions, TMD are also associated with numerous comorbidities such as anxiety, poor sleep quality, and depression [6]. Although associations with TMD have been extensively described, factors related to longitudinal variations in TMD, such as symptom onset and remission, are less explored. This knowledge gap constitutes a clinical challenge because such factors may play an important role in understanding individual prognosis and affect clinical decision making. The painful aspect of TMD is what most clearly affects the lives of individuals with TMD [5], so in this study, we will focus especially on painful TMD, i.e., TMD pain.

In a previous study, we demonstrated that the prevalence of chronic orofacial pain has increased with time, signifying the importance of understanding factors associated with the persistence and remission of TMD symptoms. In addition, the OPPERA project identified somatic symptoms, deteriorating sleep quality, and comorbid health conditions as associated with TMD‐pain onset [7]. Regarding TMD pain, previous studies have primarily focused on psychological and biological factors [8, 9], although social determinants, i.e., non‐medical life conditions that can affect health outcomes, have also been suggested to be of potential importance [10]. In this regard, recent research suggests that social determinants may affect both pain perception and prognosis [11]. Therefore, an improved understanding of the social context could provide important information for clinical decision making and for managing TMD pain. The relationship between TMD pain and its potential social determinants has, however, been sparsely studied.

We recently demonstrated that TMD is associated with being on long‐term sick leave and experiencing reduced health‐related quality of life (HRQoL) [12]. In this context, sick leave can be viewed as a social determinant shaping health outcomes, as previous research has shown an increased risk of social withdrawal and other negative consequences [13, 14, 15]. Still, the longitudinal aspect of the onset and remission of TMD pain in relation to sick leave and HRQoL has not been explored. Moreover, and in line with previous research on gender differences in pain, we found that, compared with men, women experienced TMD‐pain onset more frequently and were less likely to recover from it [16]. However, it is not known what factors are related to gender differences in long‐term variations in TMD pain. To bridge these existing knowledge gaps, our aim was to assess whether a history of sick leave and reduced HRQoL, respectively, are associated with TMD‐pain onset and remission. In addition, we also aimed to assess whether any such associations differ depending on gender, level of education, physical activity, and age.

## 2 Methods

This study was approved by the Regional Ethical Review Board at Umeå University, Sweden (ref. no. 2019–04534). The STROBE statement on reporting results was followed [17].

### 2.1 Study Design

**FIGURE 1:**
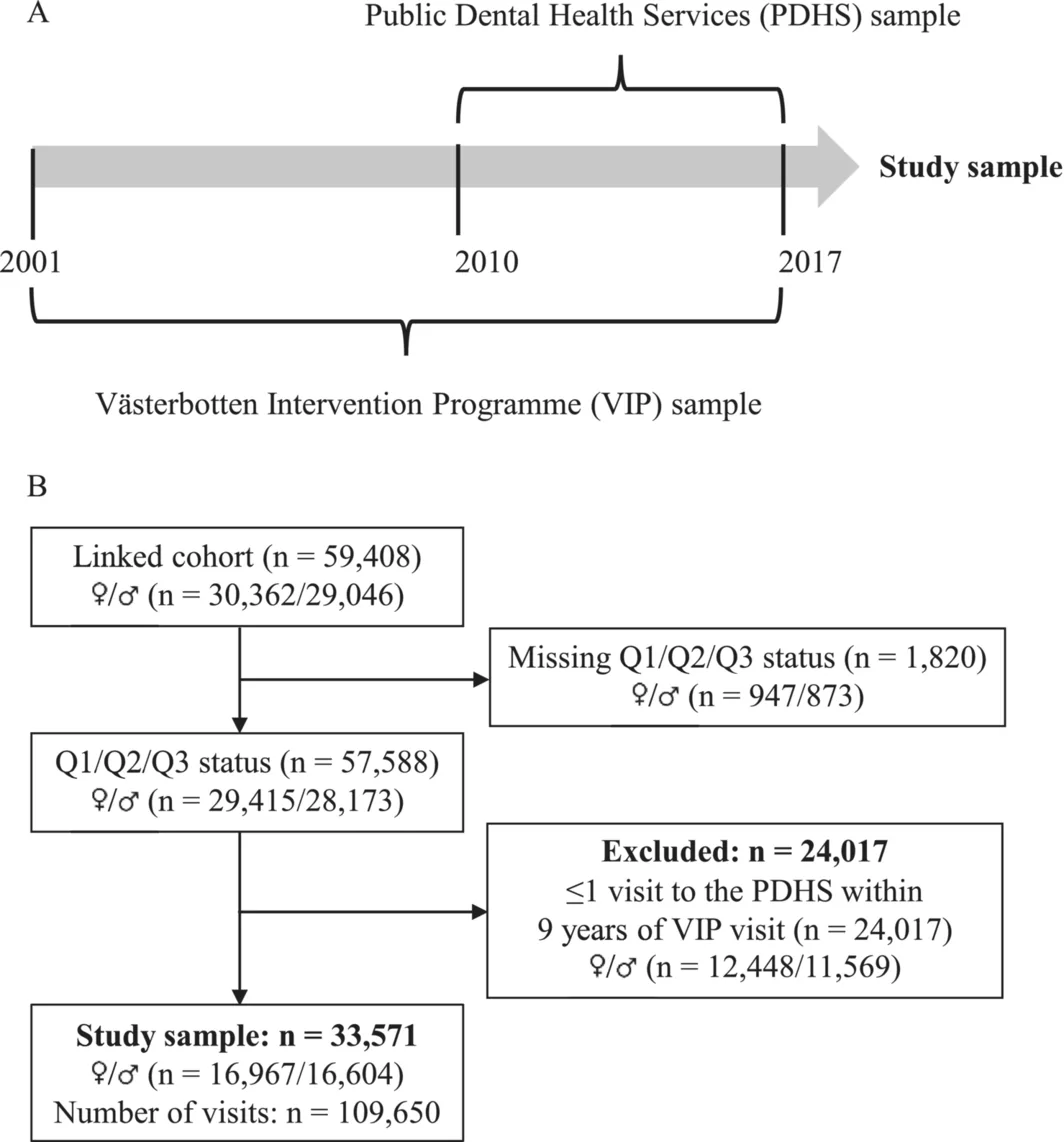
Flow chart, step‐by‐step, of inclusion and exclusion criteria. (A) Timeline of data sources for the study sample: The VIP data were collected up to 9 years before the PDHS data were collected; older data were excluded. (B) The step‐by‐step process of inclusion in and exclusion from the study sample.

In this cohort study, longitudinal data on TMD pain status were collected from regular dental check‐ups. Data on history of sick leave and HRQoL, together with covariates, were collected via participation in an intervention programme (Figure 1).

### 2.2 Study Population and Setting

Data on TMD pain were retrieved from the Public Dental Health Services (PDHS) in Västerbotten County, northern Sweden, based on three validated screening questions for TMD (3Q/TMD) [18], phrased as follows:

Do you have pain in the temple, jaw, or jaw joint once a week or more?

Do you have pain when you open your mouth or chew, once a week or more?

Does your jaw lock, or become stuck, once a week or more?

An affirmative answer to one of the pain questions (Q1 and Q2) has been shown to identify the most common TMD‐pain diagnoses in the general population [18]. Thus, the PDHS sample yielded longitudinal data on TMD pain in all individuals who made repeated visits to the PDHS between 2010 and 2017, when the 3Q/TMD questions were mandatory. All individuals who underwent at least two routine dental check‐ups and answered the 3Q/TMD questions were eligible for linkage to cross‐sectional data from the Västerbotten Intervention Programme (VIP), provided they were not older than 74 years at the time of their PDHS visits and had made a VIP visit up to 9 years before their PDHS visits (Figure 1A). Thus, individuals making only a single PDHS visit were excluded (Figure 1B). Västerbotten residents are invited for voluntary health screening primarily at 40, 50, and 60 years of age. However, occasionally individuals outside these target age groups have also been invited, resulting in a limited number of individuals younger than 40 years of age being included in the study cohort [12]. Approximately 60% of the eligible population choose to participate, and there are only minor differences in socioeconomic and education levels between participants and non‐participants in Västerbotten [19, 20]. Further details on this cohort are provided elsewhere [12].

### 2.3 Variables

**FIGURE 2:**
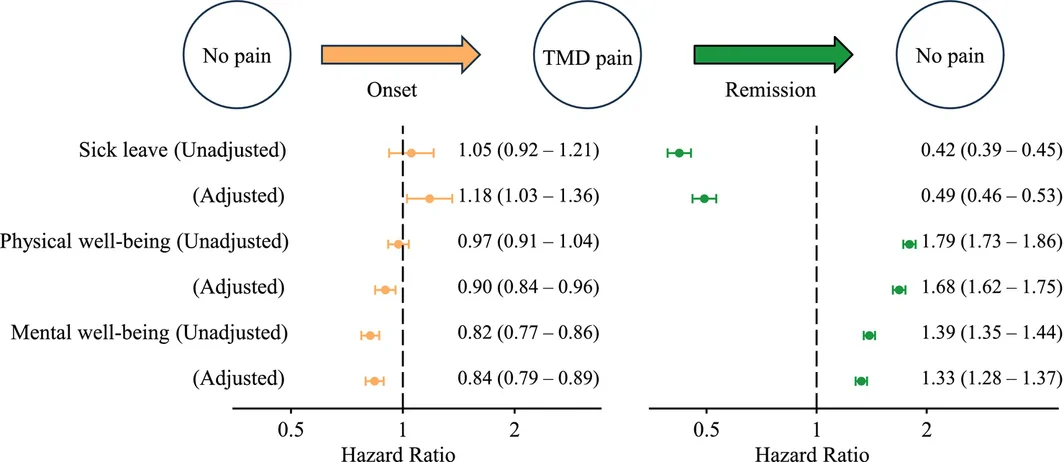
Hazard ratios (HRs) for transition rates between temporomandibular (TMD) pain and no pain, based on Markov multi‐state models. Above, the transition between the two specified states, no pain and TMD pain; the orange arrow (left‐hand side) represents pain onset, and the green arrow (right‐hand side) represents pain remission. Below, the HRs for transition rates between TMD pain and no pain, based on Markov multi‐state models; colour coding as shown above. The bars indicate 95% confidence intervals for the association between TMD pain and sick leave, physical well‐being (Physical Component Summary), and mental well‐being (Mental Component Summary), respectively. For sick leave, the HR compares individuals with a reported history of prolonged sick leave with those without such a history. For physical well‐being and mental well‐being, the HR represents the expected change in HR for a 10‐unit increase in the respective measure.

The outcome was TMD pain, defined as an affirmative answer to at least one of the two questions on pain (Q1 or Q2), whereas no pain was defined as negative answers to both Q1 and Q2, regardless of the response to Q3. Pain onset was defined as a transition from no pain to TMD pain, and pain remission as a transition from TMD pain to no pain (Figure 2). The independent variables of interest were sick leave, defined as self‐reported sick leave for 6 consecutive months or more, and HRQoL, as measured by the Swedish version of the Short Form 36 Health Survey Questionnaire (SF‐36). The SF‐36 is an instrument used to measure health status at both the individual and population levels, and as such it is applicable for both research and clinical purposes [21]. The SF‐36 consists of 36 questions that cover eight domains and also features two summary scores, the Physical Component Summary (PCS) and Mental Component Summary (MCS). PCS and MCS were used to measure HRQoL, with higher scores representing better HRQoL. We used PCS and MCS as indicators of physical and mental well‐being, respectively.

Self‐reported physical activity was determined using the Cambridge Index [22], an instrument measuring physical activity at work and during recreational activities. The four original categories (i.e., inactive, moderately inactive, moderately active, and active) were dichotomised to inactive (i.e., sedentary job and no physical recreational activity) and active. The highest educational level was defined as university education or no university/no information. Age at visit to the PDHS and time between the VIP visit and PDHS visit were used as covariates or stratification variables.

All explanatory variables, except for age and year of PDHS visit, were collected from the VIP. These variables were time varying for each individual, such that each TMD observation was linked to the individual's most recent available VIP visit in relation to the PDHS visit (up to a maximum of 9 years before and at the latest within the same year as the PDHS visit).

### 2.4 Statistical Methods

Using Markov multi‐state models with visiting year as the time scale, we analysed the association between transitions to and from TMD pain and each of the following independent variables: sick leave, physical well‐being, and mental well‐being. Visiting year was chosen to provide an implicit adjustment for possible trends in pain onset and remission over calendar time. This decision was based on previously observed time trends in the data during the study period [1]. All models were adjusted for age, time between VIP and PDHS visits, and gender, except when stratifying for gender. To account for nonlinearity, age was adjusted using restricted cubic splines with four knots placed at the 5th, 35th, 65th, and 95th percentiles of the age distribution. For each independent variable, interactions with gender, physical activity, and education were explored in three separate models. Individuals were considered at risk of transitioning between states from their first recorded visit to the PDHS until their last recorded visit. As the PDHS data only capture each individual's TMD‐pain status at their PDHS visits, but not the exact time point of the transition, interval censoring was employed [23].

Furthermore, subgroup analyses, stratified by gender, education, and physical activity, were performed. Finally, a subgroup analysis based on age was performed using the following age groups: ≤ 44, 45–60, and > 60 years of age. The ≤ 44‐year category was used in the OPPERA project [24]. Additionally, in the three separate models, we explored interactions between age group, on one hand, and sick leave, physical well‐being, and mental well‐being, on the other. Each Markov multi‐state model estimated two different hazard ratios (HRs) with corresponding 95% confidence intervals (CIs): one for the rate of transition from no pain to TMD pain (i.e., rate of pain onset) and one for the transition from TMD pain to no pain (i.e., rate of pain remission). For sick leave, the HRs compare the rates of pain onset and remission between the individuals reporting sick leave with the rates among those not reporting it, with an HR > 1 indicating a higher rate in individuals reporting sick leave. For physical and mental well‐being, which are continuous variables, the HR represents the (multiplicative) change in rate per unit increase in physical well‐being and mental well‐being. To improve the readability of the CIs, the physical health and mental health summary scores were divided by a factor of 10, i.e., the HR represents the expected change in HR for a 10‐unit increase in the respective measure.

In a complementary sensitivity analysis, we performed multiple imputation to assess the effect of missing values on sick leave status, physical well‐being, and mental well‐being (see Supporting Information).

Analyses and calculations were conducted in R [25], the multi‐state models were fitted using the ‘msm’ package [23], and splines were computed using the ‘rms’ package [26].

## 3 Results

### 3.1 Study Sample Characteristics

In total, 33 571 individuals were included, contributing a total of 109 650 PDHS visits. Most of the individuals made between two and four PDHS visits during the study period (Table 1), with the number of visits equally distributed between women (50.6%) and men. At inclusion (i.e., at first PDHS visit), 6.9% of the women and 2.5% of the men reported TMD pain. The period prevalence of pain, defined as TMD pain at any time during the study period, was higher among women than men (11.6% vs. 4.5%). During the study period, pain onset was reported by 345 individuals (250 women vs. 95 men) and pain remission by 253 individuals (183 women vs. 70 men). The mean age at first PDHS visit was 52 years for both women and men (Table 1). At first visit, the youngest individual was aged 23 years and the oldest 74.

**TABLE 1:** Sample characteristics, stratified by men and women.

Variable	Gender		
	Overall *n* = 33 571	Men *n* = 16 604	Women *n* = 16 967
TMD pain at inclusion	1593 (4.7%)	416 (2.5%)	1177 (6.9%)
Mean (Q1, Q3) age at first visit	52 (43, 60)	52 (43, 60)	52 (43, 60)
University education	9854 (29%)	3816 (23%)	6038 (36%)
Physically inactive^a^	5341 (16%)	2780 (17%)	2561 (15%)
Mental well‐being^b^ ^,^ ^c^	51 (48, 56)	52 (49, 56)	50 (47, 56)
Physical well‐being^b^ ^,^ ^c^	50 (46, 56)	51 (48, 56)	49 (44, 56)
Sick leave^d^	6899 (21%)	2619 (16%)	4280 (26%)
Number of visits			
2	9624 (29%)	4715 (28%)	4909 (29%)
3	11 291 (34%)	5627 (34%)	5664 (33%)
4	8154 (24%)	4112 (25%)	4042 (24%)
5	3365 (10%)	1628 (9.8%)	1737 (10%)
6	897 (2.7%)	414 (2.5%)	483 (2.8%)
7	214 (0.6%)	97 (0.6%)	117 (0.7%)
8	26 (< 0.1%)	11 (< 0.1%)	15 (< 0.1%)
Number of visits with reported TMD pain^e^			
0	30 938 (92%)	15 873 (96%)	15 065 (89%)
1	945 (2.8%)	299 (1.8%)	646 (3.8%)
2	782 (2.3%)	206 (1.2%)	576 (3.4%)
3	520 (1.5%)	134 (0.8%)	386 (2.3%)
4	255 (0.8%)	68 (0.4%)	187 (1.1%)
5	106 (0.3%)	22 (0.1%)	84 (0.5%)
6	21 (< 0.1%)	2 (< 0.1%)	19 (0.1%)
7	4 (< 0.1%)	0 (0%)	4 (< 0.1%)

Abbreviations: PDHS, Public Dental Health Services; Q, Quartile; TMD, Temporomandibular disorder.

^a^ Information regarding physical activity was missing for 205 men and 241 women.

^b^ Mental well‐being assessed with the Mental Component Summary and physical well‐being assessed with the Physical Component Summary.

^c^ Information regarding the Mental Component Summary and the Physical Component Summary was missing for 2263 men and 2304 women, respectively.

^d^ Information regarding sick leave was missing for 569 men and 724 women.

^e^ No individual with TMD pain made eight visits to the PDHS.

At inclusion, women had higher levels of university education (36% vs. 23%) and sick leave (26% vs. 16%) than did men. Women and men had similar values for HRQoL and physical inactivity.

Individuals with sick leave reported more TMD pain at inclusion (9.0% vs. 3.5%, Table 2). Both individuals with and without sick leave made a mean number of three visits during the study period, although the average age was higher for those with sick leave (54 vs. 51 years).

**TABLE 2:** Sample characteristics across reported history of prolonged sick leave at inclusion, stratified by gender.

Characteristic	Information on history of prolonged sick leave at inclusion								
	Reported			Not reported			Missing		
	Overall *n* = 6899	Men *n* = 2619	Women *n* = 4280	Overall *n* = 25 379	Men *n* = 13 416	Women *n* = 11 963	Overall *n* = 1293	Men *n* = 569	Women *n* = 724
TMD pain at inclusion	618 (9.0%)	115 (4.4%)	503 (12%)	892 (3.5%)	281 (2.1%)	611 (5.1%)	83 (6.4%)	20 (3.5%)	63 (8.7%)
Mean (Q1, Q3) age at first visit	54 (46, 61)	55 (48, 62)	54 (46, 61)	51 (42, 59)	51 (42, 59)	51 (42, 58)	54 (46, 62)	53 (45, 61)	54 (46, 62)
University education	1658 (24%)	422 (16%)	1236 (29%)	8035 (32%)	3332 (25%)	4703 (39%)	161 (12%)	62 (11%)	99 (14%)
Physically inactive	1242 (18%)	507 (20%)	735 (17%)	3887 (15%)	2173 (16%)	1714 (14%)	212 (21%)	100 (24%)	112 (20%)
Mental well‐being^a^	48 (42, 56)	49 (44, 56)	47 (41, 56)	51 (49, 56)	52 (50, 56)	51 (48, 56)	50 (46, 57)	50 (46, 57)	49 (45, 57)
Physical well‐being^b^	42 (34, 52)	43 (35, 52)	42 (34, 51)	52 (49, 56)	52 (50, 56)	51 (49, 56)	45 (38, 54)	47 (42, 55)	44 (36, 53)
Number of PDHS visits									
2	1809 (26%)	655 (25%)	1154 (27%)	7404 (29%)	3873 (29%)	3531 (30%)	411 (32%)	187 (33%)	224 (31%)
3	2230 (32%)	853 (33%)	1377 (32%)	8659 (34%)	4588 (34%)	4071 (34%)	402 (31%)	186 (33%)	216 (30%)
4	1777 (26%)	715 (27%)	1062 (25%)	6093 (24%)	3282 (24%)	2811 (23%)	284 (22%)	115 (20%)	169 (23%)
5	791 (11%)	297 (11%)	494 (12%)	2438 (9.6%)	1278 (9.5%)	1160 (9.7%)	136 (11%)	53 (9.3%)	83 (11%)
6	226 (3.3%)	72 (2.7%)	154 (3.6%)	627 (2.5%)	323 (2.4%)	304 (2.5%)	44 (3.4%)	19 (3.3%)	25 (3.5%)
7	61 (0.9%)	26 (1.0%)	35 (0.8%)	139 (0.5%)	63 (0.5%)	76 (0.6%)	14 (1.1%)	8 (1.4%)	6 (0.8%)
8	5 (< 0.1%)	1 (< 0.1%)	4 (< 0.1%)	19 (< 0.1%)	9 (< 0.1%)	10 (< 0.1%)	2 (0.2%)	1 (0.2%)	1 (0.1%)
Number of visits with reported TMD pain^c^									
0	5967 (86%)	2424 (93%)	3543 (83%)	23 799 (94%)	12 910 (96%)	10 889 (91%)	1172 (91%)	539 (95%)	633 (87%)
1	315 (4.6%)	76 (2.9%)	239 (5.6%)	597 (2.4%)	215 (1.6%)	382 (3.2%)	33 (2.6%)	8 (1.4%)	25 (3.5%)
2	255 (3.7%)	56 (2.1%)	199 (4.6%)	487 (1.9%)	141 (1.1%)	346 (2.9%)	40 (3.1%)	9 (1.6%)	31 (4.3%)
3	204 (3.0%)	38 (1.5%)	166 (3.9%)	286 (1.1%)	85 (0.6%)	201 (1.7%)	30 (2.3%)	11 (1.9%)	19 (2.6%)
4	93 (1.3%)	16 (0.6%)	77 (1.8%)	148 (0.6%)	51 (0.4%)	97 (0.8%)	14 (1.1%)	1 (0.2%)	13 (1.8%)
5	51 (0.7%)	8 (0.3%)	43 (1.0%)	53 (0.2%)	13 (< 0.1%)	40 (0.3%)	2 (0.2%)	1 (0.2%)	1 (0.1%)
6	13 (0.2%)	1 (< 0.1%)	12 (0.3%)	7 (< 0.1%)	1 (< 0.1%)	6 (< 0.1%)	1 (< 0.1%)	0 (0%)	1 (0.1%)
7	1 (< 0.1%)	0 (0%)	1 (< 0.1%)	2 (< 0.1%)	0 (0%)	2 (< 0.1%)	1 (< 0.1%)	0 (0%)	1 (0.1%)

Abbreviations: PDHS, Public Dental Health Services; Q, Quartile; TMD, Temporomandibular disorder.

^a^ Mental well‐being assessed with the Mental Component Summary.

^b^ Physical well‐being assessed with the Physical Component Summary.

^c^ No individual with TMD pain made eight visits to the PDHS.

The imputed analyses were in line with the results presented above, albeit with a higher educational level among individuals reporting sick leave status and HRQoL (Supporting Information S2 and S3).

### 3.2 Associations

Individuals with missing information regarding sick leave (*n* = 1293) and HRQoL (*n* = 4567) were omitted from the corresponding analyses.

When adjusting for age at PDHS visit, time between VIP and PDHS visits, and gender, sick leave was associated with higher rates of TMD‐pain onset (HR: 1.18, CI: 1.03–1.36) (Figure 2). Better physical well‐being and mental well‐being were associated with lower rates of pain onset (HR per 10‐unit increase: 0.90, CI: 0.84–0.96, and 0.84, CI: 0.79–0.89, respectively). Furthermore, sick leave was associated with a lower rate of pain remission (HR: 0.49, CI: 0.46–0.53), whereas better physical well‐being and mental well‐being were associated with a higher rate of pain remission (HR: 1.68, CI: 1.62–1.75, and 1.33, CI: 1.28–1.37, respectively).

**FIGURE 3:**
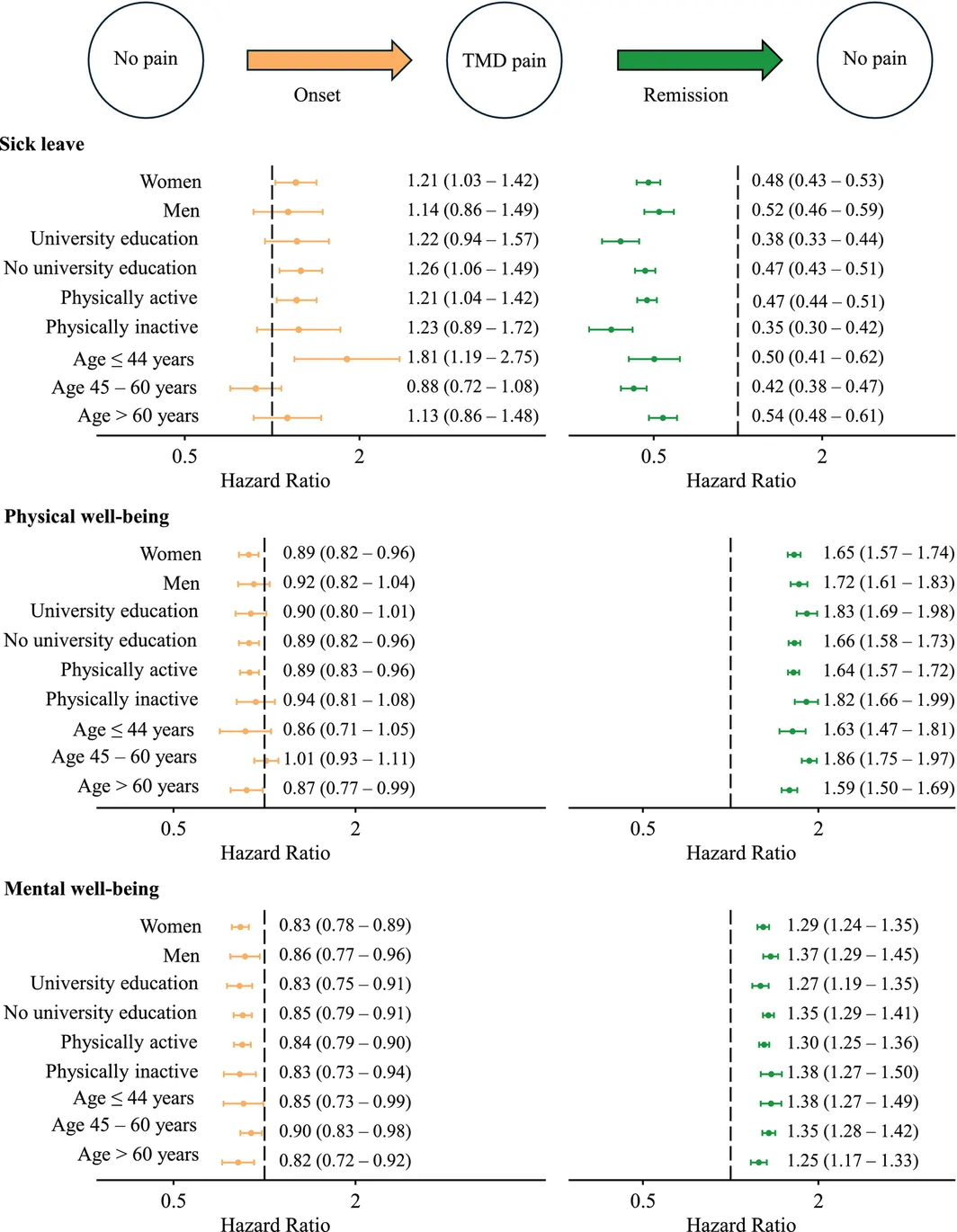
Subgroup analysis stratified by gender, education, physical activity, and age. Hazard ratios (HRs) for transition rates between temporomandibular (TMD) pain and no pain, based on Markov multi‐state models. Above, the transition between the two specified states, no pain and TMD pain; the orange arrow (left‐hand side) represents pain onset, and the green arrow (right‐hand side) represents pain remission. Below, the HRs for transition rates between TMD pain and no pain, based on Markov multi‐state models; colour coding as shown above. The bars indicate 95% confidence intervals for the association between TMD pain and sick leave, physical well‐being, and mental well‐being, respectively. For sick leave, the HR compares individuals with a reported history of sick leave with those without such a history. For physical well‐being and mental well‐being, the HR represents the expected change in HR for a 10‐unit increase in the respective measure.

For pain onset, all subgroup analyses produced results similar to those for the total cohort (Figure 3). For pain remission, however, there were a few significant interactions. The interaction between sick leave and physical activity was significant (*p* = 0.002). For individuals with sick leave, the transition rates were lower among individuals who were also physically inactive than among individuals who were physically active. The interaction between sick leave and education was significant (*p* = 0.033), with sick leave being associated with lower transition rates for individuals with a university education than for individuals without a university education. Moreover, the interaction between sick leave and age group was significant (*p* = 0.020), with individuals on sick leave aged 45–60 years having lower transition rates than did individuals older than 60 years.

In addition, the interaction between physical well‐being and physical activity was significant (*p* = 0.026), with better physical well‐being associated with higher transition rates among physically inactive individuals than among physically active individuals. Finally, the interaction between physical well‐being and education was significant (*p* = 0.049), with better physical well‐being being associated with higher transition rates among individuals with a university education than among individuals without a university education. Collectively, and even though there were some significant differences between subgroups, the HRs were consistent in direction and similar in size. Thus, we found no evidence for heterogeneity in the data.

## 4 Discussion

The main finding of this study is that individuals with better mental well‐being (MCS) and physical well‐being (PCS) were less likely to report TMD‐pain onset. In addition, individuals with better mental well‐being and physical well‐being were more likely to report TMD‐pain remission than were individuals with worse well‐being. Emphasising the importance of the association between comorbidities and TMD pain, individuals with a history of sick leave were more likely to report TMD‐pain onset and less likely to report remission from TMD pain than were individuals without a history of sick leave.

### 4.1 Social Determinants

Given the multifactorial nature of chronic pain, knowledge of social determinants such as socioeconomic status is imperative in order to understand pain variations. Building on such a conceptual understanding of pain, a recent umbrella review showed that lower socioeconomic status was associated with increased odds of developing chronic musculoskeletal pain [27]. In this context, sick leave can be regarded as a social determinant of health, since sick leave can reflect work‐related strain, socioeconomic status, and health status [28]. Our finding of the association between sick leave and less pain remission is in line with a register study from Sweden showing that TMD was associated with sick leave in a referred patient sample [29]. In this context, we view education as a proxy for socioeconomic status [30]. By stratifying on education, we can conclude that the socioeconomic differences in the associations are small and we note that the HRs are consistent in direction, making the results more generalisable and transferable to other settings. Given the burden of pain, our findings suggest that sick leave not only is linked to specialist‐treated TMD but should also be considered during initial assessment in general dental practice.

Regarding physical activity, evidence supports a positive relationship between being physically active and higher socioeconomic status, illustrating yet another example of a social determinant of health [31]. Although a recent study found no direct evidence of an association between TMD and physical activity [32], it is still important to evaluate this possible relationship. The results were similar for physically active and inactive individuals, indicating that it is not the physical activity itself that explains these differences. The significant interaction between sick leave and physical activity could indicate that individuals with a physically active life are better suited to cope with sick leave, or that these individuals go on sick leave for other reasons. Overall, in line with the biopsychosocial model, our findings reinforce the importance of incorporating factors beyond the purely biological in pain assessment.

### 4.2 Health‐Related Quality of Life

Previous studies have shown that TMD pain is associated with worse HRQoL [5]. Our results indicate that individuals with better physical well‐being experience pain remission more frequently and onset less frequently. One possible explanation is that individuals with better physical well‐being likely have fewer somatic symptoms and comorbidities attributed to TMD [6], which in turn could ease recovery and build resilience. Moreover, TMD often coexists with symptoms such as headache, upset stomach, and sore muscles [33, 34], which could affect both the duration and quality of sleep, thereby preventing physical and mental recuperation.

Mental well‐being is associated with chronic pain in general [35] and with TMD pain more specifically [7]. However, this association has not been studied in relation to pain onset and remission. We found that better mental well‐being was associated with less frequent onset and more frequent remission of TMD pain. As such, individuals with better mental well‐being may be more mentally robust and have better coping abilities. This may be related to lower degrees of worrying and pain catastrophising [36]. Self‐efficacy is imperative for the outcome of pain rehabilitation in general [37] and for TMD more specifically [38]. Self‐management is therefore a key element of TMD treatment, advocated in the national guidelines for TMD treatment in Sweden [39] and the UK [40]. Since poorer mental well‐being can affect self‐efficacy and adherence, our results illustrate the importance of a holistic approach to treating TMD pain in general dental practice.

### 4.3 Socio‐Demographic Factors

Despite gender differences in pain, our findings indicate that the same factors are associated with TMD‐pain onset and remission in both men and women. Age‐ and gender‐related differences can be caused by biological factors (e.g., tissue response and the body's ability to heal) and social conditions over the life cycle. We used a biological rationale for the age categorisation, building on findings suggesting that ageing happens in two bursts: at around 44 and 60 years [41].

Concerning age differences, our findings indicate that the factors associated with TMD‐pain onset and remission were similar in all age groups. However, the effect of sick leave in the onset phase seemed to differ across age groups. This should be interpreted with caution since the CI for individuals younger than 44 years was relatively wide, and there was no significant interaction between sick leave and age group.

As we do not know when the sick leave occurred, it is possible that the sick leave happened longer ago in the older age groups than in the youngest group, possibly weakening the association. It is also possible that in older sickly individuals the onset of illness happened earlier, so the reduction in general health had already happened. Overall, this reinforces our findings that sick leave, physical well‐being, and mental well‐being are all associated with TMD‐pain remission, emphasising that a holistic approach is needed in TMD‐pain assessment and management for both men and women across all age groups.

This study is descriptive in nature, and its findings should be interpreted accordingly. Associations were estimated with limited adjustment to facilitate interpretation, as recommended in descriptive epidemiology [42]. Consequently, no causal inferences can be drawn. However, the findings may motivate further research into underlying pathways and inform the development of targeted prevention efforts. Further building on the descriptive and exploratory approach, we stratified the analysis and evaluated interactions to explore heterogeneity across subgroups. Interaction analyses often have limited statistical power, and no adjustments for multiple comparisons were made; nevertheless, we still deemed these analyses relevant to confirm that the associations were consistent across the population studied.

As the exact time point of pain onset in relation to the pre‐existing conditions was unknown, we regard the independent variables as indicators of previous health status. In this context, a history of sick leave may be viewed as a marker of vulnerability, reflecting not only social factors but also poorer health in general.

In this study, we limited the number of covariates included in our models to ease interpretation and avoid overadjustment. However, given the complex interplay between TMD pain, well‐being, and sick leave, further analyses also taking into account comorbidities would be relevant in order to clarify underlying patterns. Consideration of comorbidities was outside the scope of this study but represents an important direction for future studies.

### 4.4 Strengths and Limitations

The large sample size is a major strength, allowing associations to be estimated with good precision and good generalisability. The study is further strengthened by the good coverage and low socioeconomic bias of the VIP [19]. The use of longitudinal data is another strength, since the studied cohort is one of few permitting the estimation of factors associated with both pain onset and remission. Moreover, the prevalence of TMD pain in this linked cohort is in line with the expected prevalence in the general population [2]. Regarding representativeness, we have previously shown that the differences in visiting frequency among community subgroups as a potential factor affecting selection bias did not influence the prevalence [12]. Interval censoring was employed to address the fact that only the TMD‐pain status at the time of PDHS visits and not the exact time points of transitions between TMD‐pain conditions were known. This reduces the risk of bias versus assuming that an individual would remain in the same state until a transition is observed.

In this study, TMD pain was assessed at discrete PHDS visits using the 3Q/TMD. The 3Q/TMD is a validated instrument for assessing TMD pain; a positive answer to at least one of the two questions on pain (Q1 or Q2) has demonstrated high sensitivity (≈0.82) and specificity (≈0.87) [18]. As in any epidemiologic study, there is a risk of misclassification bias. However, the misclassification is unlikely to differ markedly by sick leave status, mental well‐being, or physical well‐being alone. Consequently, the associations presented in this study are more likely to be underestimated than overestimated.

One limitation is the time lag between the VIP and PDHS visits, which was set to a maximum of 9 years. The potential influence of sick leave, mental well‐being, and physical well‐being may be diminishing with time. Therefore, we used the time between the VIP visit and PHDS visit in the presented results to mitigate this. The large number of individuals aged 40–70 years makes the estimates for this group precise, whereas conclusions outside of this age range should be made with caution. However, given that nearly 80% of the adults in Västerbotten County regularly attend dental check‐ups and that about half of these check‐ups occur at the PDHS, in combination with the extensive coverage and low socioeconomic bias of the VIP [19, 43], we consider our sample to be representative of the general population of Västerbotten County aged 40 years and above.

Participants who made only one PDHS visit during the follow‐up period were excluded. The magnitude and direction of possible selection bias from this exclusion are difficult to assess, but the homogenous estimates of HRs for transitions in both directions across the investigated subgroups of the cohort suggest that the impact is small. Finally, individuals with missing data on the self‐reported independent variables were omitted from the corresponding analysis, reducing the sample size; given the overall large sample size, we regard the impact of this as small but not negligible.

## 5 Conclusions

Sick leave, physical well‐being, and mental well‐being are all associated with TMD‐pain remission. Therefore, these may be factors to consider in clinical decision making and management, emphasising the importance of applying a holistic approach to pain assessment. In addition, individuals with better mental well‐being and physical well‐being less often reported TMD‐pain onset and individuals with sick leave reported onset more often, indicating that these could be factors to consider when designing preventative strategies, not only for general health but also for pain prevention.

## Author Contributions

**S. Vallin:** contributed to the design and interpretation, performed the analysis, drafted and critically revised the manuscript. **P. Liv:** contributed to the conception, design, analysis, and interpretation, critically revised the manuscript. **B. Häggman‐Henrikson:** contributed to the interpretation and critically revised the manuscript. **C. M. Visscher:** contributed to the interpretation and critically revised the manuscript. **F. Lobbezoo:** contributed to the interpretation and critically revised the manuscript. **A. Lövgren:** contributed to the conception and design, contributed to the data acquisition and interpretation, helped draft and critically revise the manuscript. All authors gave their final approval and agreed to be accountable for all aspects of the work.

## Funding

This study was supported through a regional agreement between Umeå University and Västerbotten County Council (ALF and TUA).

## Conflicts of Interest

The authors declare no conflicts of interest.

## Supporting information


**Method S1:** Supporting Information.

**Figure S1:** Analysis including participants with missing values for sick leave, physical well‐being, and mental well‐being, with missing data imputed using multiple imputation. Hazard ratios (HRs) for transition rates between temporomandibular (TMD) pain and no pain, based on Markov multi‐state models. Above, the transition between the two specified states, no pain and TMD pain; the orange arrow (left‐hand side) represents pain onset, and the green arrow (right‐hand side) represents pain remission. Below, the HRs for transition rates between TMD pain and no pain, based on Markov multi‐state models; colour coding as shown above. The bars indicate 95% confidence intervals for the association between TMD pain and sick leave, physical well‐being (Physical Component Summary), and mental well‐being (Mental Component Summary), respectively. For sick leave, the HR compares individuals with a reported history of prolonged sick leave with those without such a history. For physical well‐being and mental well‐being, the HR represents the expected change in HR for a 10‐unit increase in the respective measure.

**Table S1:** Sample characteristics by availability of health‐related quality of life, stratified by gender.

## Data Availability

The data supporting the findings of this study are available from Region Västerbotten. Access to the data is restricted, as the data were used under licence for the current study and are therefore not publicly available. The data are, however, available from the corresponding authors upon reasonable request and with the permission of Region Västerbotten.
