# Supplementary material for: Mental Well‐Being Is Associated With Temporomandibular Disorder Pain Onset and Remission
Source: J Oral Rehabil. 2026 Jun 18;53(10):1878–88. doi: 10.1111/joor.70236 (PMC13583372; doi:10.1111/joor.70236)
Supplement: Supplementary file 1 [file JOOR-53-1878-s002.pdf]

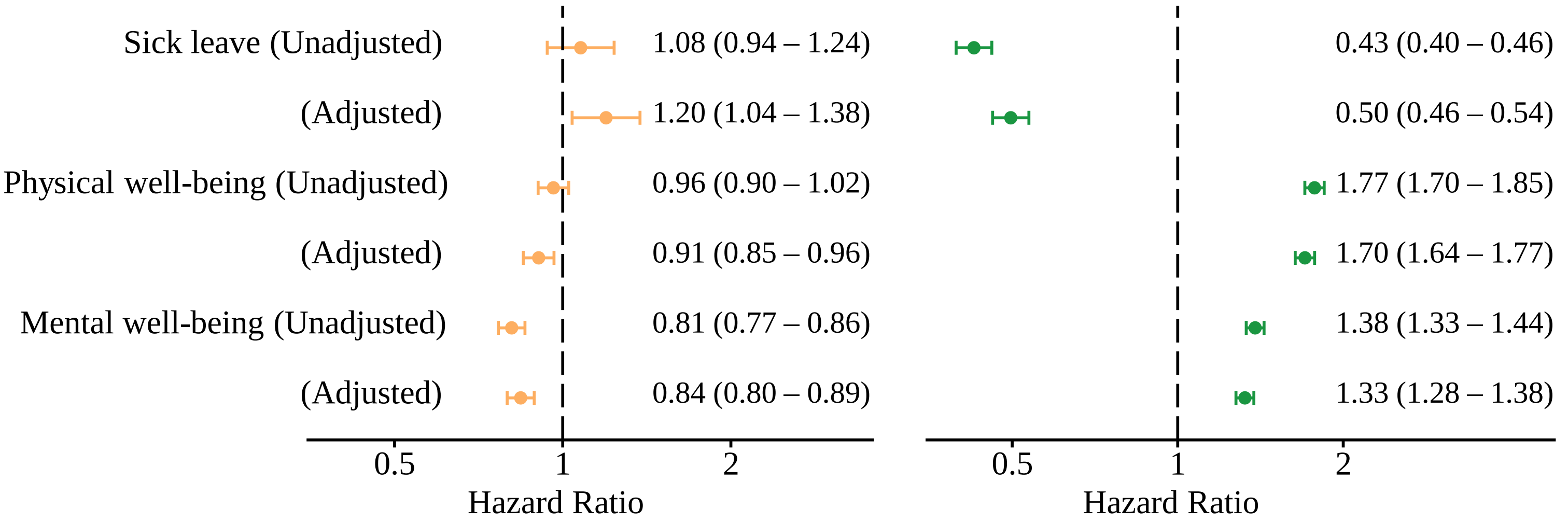

Sick leave (Unadjusted)
(Adjusted)
Physical well-being (Unadjusted)
(Adjusted)
Mental well-being (Unadjusted)
(Adjusted)
1.08 (0.94 – 1.24)
1.20 (1.04 – 1.38)
0.96 (0.90 – 1.02)
0.91 (0.85 – 0.96)
0.81 (0.77 – 0.86)
0.84 (0.80 – 0.89)
0.43 (0.40 – 0.46)
0.50 (0.46 – 0.54)
1.77 (1.70 – 1.85)
1.70 (1.64 – 1.77)
1.38 (1.33 – 1.44)
1.33 (1.28 – 1.38)
0.5
1
2
Hazard Ratio
0.5
1
2
Hazard Ratio
