# Supplementary material for: Mental Well‐Being Is Associated With Temporomandibular Disorder Pain Onset and Remission
Source: J Oral Rehabil. 2026 Jun 18;53(10):1878–88. doi: 10.1111/joor.70236 (PMC13583372; doi:10.1111/joor.70236)
Supplement: Supplementary file 2 [file JOOR-53-1878-s001.docx]

Multiple imputation was performed using predictive mean matching with 10 iterations and 10 donors to impute 20 datasets. The missing values were imputed using the following variables: age, gender, BMI, TMD pain (at any time during the study period), smoking status, Cambridge Index, systolic and diastolic blood pressure, cholesterol, and diabetes status.
In addition, when imputing sick leave, physical well-being and mental well-being were used, and when imputing physical well-being and mental well-being, sick leave was used.
The parameter estimates and variance were extracted from each imputed dataset and pooled using Rubin’s rule. The imputation and the implementation of Rubin’s rule were conducted using the “mice” package [1]

1. van Buuren S, Groothuis-Oudshoorn K. mice: Multivariate Imputation by Chained Equations in R. Journal of Statistical Software 2011;**45**(3):1-67 doi: 10.18637/jss.v045.i03.
