# Supplementary material for: Mental Well‐Being Is Associated With Temporomandibular Disorder Pain Onset and Remission
Source: J Oral Rehabil. 2026 Jun 18;53(10):1878–88. doi: 10.1111/joor.70236 (PMC13583372; doi:10.1111/joor.70236)
Supplement: Supplementary file 3 [file JOOR-53-1878-s003.docx]

**Supplementary table.** Sample characteristics by availability of health-related quality of life, stratified by gender

**Information on health-related quality of life (SF-36)**

|  | **Reported** | | | **Missing** | | |  |
| --- | --- | --- | --- | --- | --- | --- | --- |
| **Characteristic** | **Overall**,  n = 29,004 | **Men**,  n = 14,341 | **Women**,  n = 14,663 | **Overall**,  n = 4,567 | **Men**,  n = 2,263 | **Women**,  n = 2,304 |  |
| TMD pain at inclusion | 1,388 (4.8%) | 359 (2.5%) | 1,029 (7.0%) | 205 (4.5%) | 57 (2.5%) | 148 (6.4%) |  |
| Sick leave | 5,900 (21%) | 2,214 (16%) | 3,686 (26%) | 999 (24%) | 405 (20%) | 594 (29%) |  |
| Mean (Q1, Q3) age at first visit | 51 (42, 60) | 51 (42, 60) | 51 (42, 60) | 56 (48, 65) | 56 (48, 63) | 56 (48, 66) |  |
| University education | 8,886 (31%) | 3,448 (24%) | 5,438 (37%) | 968 (21%) | 368 (16%) | 600 (26%) |  |
| Physically inactive | 4,577 (16%) | 2,392 (17%) | 2,185 (15%) | 764 (18%) | 388 (18%) | 376 (18%) |  |
| Number of visits |  |  |  |  |  |  |  |
| 2 | 8,788 (30%) | 4,301 (30%) | 4,487 (31%) | 836 (18%) | 414 (18%) | 422 (18%) |  |
| 3 | 9,853 (34%) | 4,908 (34%) | 4,945 (34%) | 1,438 (31%) | 719 (32%) | 719 (31%) |  |
| 4 | 6,766 (23%) | 3,418 (24%) | 3,348 (23%) | 1,388 (30%) | 694 (31%) | 694 (30%) |  |
| 5 | 2,722 (9.4%) | 1,318 (9.2%) | 1,404 (9.6%) | 643 (14%) | 310 (14%) | 333 (14%) |  |
| 6 | 694 (2.4%) | 313 (2.2%) | 381 (2.6%) | 203 (4.4%) | 101 (4.5%) | 102 (4.4%) |  |
| 7 | 162 (0.6%) | 74 (0.5%) | 88 (0.6%) | 52 (1.1%) | 23 (1.0%) | 29 (1.3%) |  |
| 8 | 19 (<0.1%) | 9 (<0.1%) | 10 (<0.1%) | 7 (0.2%) | 2 (<0.1%) | 5 (0.2%) |  |
| Number of visits with reported TMD pain^*^ |  |  |  |  |  |  |  |
| 0 | 26,695 (92%) | 13,708 (96%) | 12,987 (89%) | 4,243 (93%) | 2,165 (96%) | 2,078 (90%) |  |
| 1 | 830 (2.9%) | 261 (1.8%) | 569 (3.9%) | 115 (2.5%) | 38 (1.7%) | 77 (3.3%) |  |
| 2 | 709 (2.4%) | 183 (1.3%) | 526 (3.6%) | 73 (1.6%) | 23 (1.0%) | 50 (2.2%) |  |
| 3 | 452 (1.6%) | 114 (0.8%) | 338 (2.3%) | 68 (1.5%) | 20 (0.9%) | 48 (2.1%) |  |
| 4 | 212 (0.7%) | 56 (0.4%) | 156 (1.1%) | 43 (0.9%) | 12 (0.5%) | 31 (1.3%) |  |
| 5 | 88 (0.3%) | 18 (0.1%) | 70 (0.5%) | 18 (0.4%) | 4 (0.2%) | 14 (0.6%) |  |
| 6 | 15 (<0.1%) | 1 (<0.1%) | 14 (<0.1%) | 6 (0.1%) | 1 (<0.1%) | 5 (0.2%) |  |
| 7 | 3 (<0.1%) | 0 (0%) | 3 (<0.1%) | 1 (<0.1%) | 0 (0%) | 1 (<0.1%) |  |
| Abbreviations: TMD, Temporomandibular disorder; PDHS, Public Dental Health Services; Q, Quartile. ^*^No individual with TMD pain made eight visits to the PDHS. | | | | | | | |
